# Mental health stigma and legal responses to defendants with mental health conditions in Bulgaria, Japan, and Norway

**DOI:** 10.3389/fpsyg.2026.1801646

**Published:** 2026-05-05

**Authors:** Linda Gröning, Slavka Dimitrova, Miki Hirano

**Affiliations:** 1 Faculty of Law, University of Bergen, Bergen, Norway; 2 Regional Centre for Research and Education in Forensic Psychiatry and Psychology, Haukeland University Hospital of Bergen, Bergen, Norway; 3 Faculty of Law, Burgas Free University, Burgas, Bulgaria; 4 Faculty of Law, Kagawa University, Takamatsu, Japan

**Keywords:** comparative law, criminal law, criminal responsibility, health law, mental health stigma, preventive measures, rehabilitation

## Abstract

This article investigates, from a legal perspective, how criminal justice systems in Bulgaria, Japan, and Norway produce mental health stigma in their legal responses to defendants with mental health conditions. Although these countries operate within distinct legal traditions and cultural contexts, they all employ special rules on criminal responsibility and special preventive measures for this group of defendants. These rules and their application in legal practice may produce stigma through the association of legal notions, such as insanity and dangerousness, with psychiatric notions of mental disorders. Moreover, in all three countries the systems for the treatment and societal re-integration of defendants subjected to special preventive measures give rise to issues of separation and structural stigma. Our investigation indicates that there are profound challenges in all three countries, but that the pathways for stigma differ. In Japan and Bulgaria, societal stigma seems to interact with legal frameworks within a collectivistic culture in ways that disadvantage defendants with mental health conditions, while in Norway stigma appears to be more subtly embedded in risk-focused policies despite strong welfare structures. Importantly for further research however, our comparative discussion suggests that stigma in this context is not merely a cultural by product but is actively produced and reinforced through legal categories, practices, and policy choices. The findings highlight the need for legal reform focused on modernising legal terminology, mitigating discriminatory associations between legal and psychiatric constructs, and strengthening community-based pathways for treatment and reintegration.

## 1 Introduction

This paper concerns the issue of how mental health stigma in society influences legal practices and especially how these practices may contribute to the production and reinforcement of stigma in society. The paper provides a comparative discussion of this issue, considering the legal systems for dealing with individuals with mental health conditions who commit criminalised actions (hereafter “defendants with mental health conditions”)^1^ in Bulgaria, Japan, and Norway. The focus is on the questions of how the legal systems in these countries apply rules on criminal responsibility and preventive measures for defendants with mental health conditions, and what challenges related to mental health stigma arise in this context.

The legal systems in Bulgaria, Japan and Norway have significantly different societal and cultural contexts. At the same time, these countries have similar structures and functions for the responses to defendants with mental health conditions. They all include special rules on criminal responsibility for these defendants, along with preventive measures—typically involving compulsory treatment and confinement—aimed at preventing reoffending and facilitating their societal reintegration. This combination of functional and structural similarity and legal-cultural diversity makes these countries’ legal systems well suited for comparative study.

We argue that there are challenges in all three countries at different levels of legal regulation and systems for preventive measures. By illuminating these challenges, we hope to contribute to increased awareness about the role of criminal law and the criminal justice system in the production and reinforcement of mental health stigma in society. Although there is extensive research on criminal justice and mental health stigma (see Section 2), legal research examining the intersection of these fields remains limited, and comparative analyses of criminal justice rules and practices are scarce. Our investigation of legal responses to defendants with mental health conditions in Bulgaria, Japan, and Norway seeks to fill this gap.

First, in Section 2, we outline the conceptual and methodological framework for our analysis. Here, we clarify how we address the concept of stigma, particularly mental health stigma, and how legal responses to defendants with mental health conditions relate to such stigma (2.1). Section 2 also sets out the approach for our comparative investigation (2.2). It should be noted at the outset, however, that this investigation is conducted from a legal doctrinal and analytical perspective rather than an empirical one. In Section 3, we outline the overall legal-cultural differences between Bulgaria, Japan, and Norway, with particular attention to the presence of mental health stigma. Building on this backdrop, Section 4 examines the legal responses to defendants with mental health conditions in the three countries. Here, we will provide an overall description of the relevant rules and systems as a basis for the subsequent comparative discussion of stigma-related challenges in Section 5. Finally, Section 6 offers reflections on the path forward, including the legal reforms that may be needed.

## 2 Conceptual and methodological framework

### 2.1 Mental health stigma in the context of criminal justice

There is extensive contemporary discourse on stigma and its definition (Andersen et al., 2022). Erving Goffman’s work remains foundational for the current social scientific discourse. He defined stigma as a “deeply discrediting” attribute and that reduces a person “from a whole and usual” individual to a tainted, discounted one” (Goffman, 1963, p. 3). Moreover, he identified three types of such stigma relating to physical deformities, character blemishes—including criminality and mental disorder—and tribal origin, such as ethnicity and religion. Crucially, he emphasised the relational nature of stigma and introduced the notion of courtesy stigma, or stigma by association. This relational dimension is particularly relevant in the context of legal responses to defendants with mental health conditions (see Sections 4, 5).

Link and Phelan later argued that the term stigma should be applied “when elements of labeling, stereotyping, separation, status loss and discrimination co-occur in a power situation that allows the component of stigma to unfold” (Link and Phelan, 2001, p. 367; Link et al., 2004 added “emotional reaction” to the 2001 definition). This framework is widely recognised, although some scholars have suggested revisions (e.g., Andersen et al., 2022). For our purposes, the components identified by Link and Phelan remain central. The criminal justice system constitutes one of the most significant power contexts in society, and—as we later discuss—legal rules on criminal responsibility involve labelling, stereotyping and an “us and them” separation—with discrimination and unequal legal treatment of defendants with mental health conditions as possible consequences. It must also be noted that these legal responses operate within asymmetrical power relations in which the stigmatized cannot reject the stigma (Jacobsen et al., 2010).

The literature recognises that stigma against people with mental health conditions occurs at several levels, including self-stigma, stigma by association, public stigma and structural stigma (see for a structured overview, Thornicroft et al., 2022). While our discussion mainly concerns public and structural stigma, we view these levels of stigma as interconnected. Public stigma is interpersonal and concerns how negative attitudes and stereotypes produce discrimination against people with mental health conditions (Link and Phelan, 2001; Thornicroft et al., 2022). Our discussion concerns discriminatory legal responses to defendants with mental health conditions, which are closely related to structural stigma—how institutions and policies perpetuate stigmatisation (Corrigan et al., 2004; Hatzenbuehler, 2016). Structural stigma also raises human rights concerns (Link and Phelan, 2001), which not least applies to our criminal justice context. The legal rules and practices we examine have been widely criticised for violating the rights of persons with disabilities (e.g., UN Doc A/HRC/10/48; Minkowitz, 2014; Gooding and Bennet, 2018).

While our focus is on mental health stigma in the criminal justice context, our discussion necessarily touches on how this intersects with criminal stigma. The group of defendants with mental health conditions that our discussion concerns are individuals who have also engaged in criminalised behaviour, and therefore face both mental health stigma and criminal stigma linked to social disapproval, discrimination, marginalisation and exclusion (e.g., Tremlin and Beazley, 2024; Moore et al., 2016; Holloway and Wiener, 2021). This dual, or intersectional stigmatisation makes them a particularly vulnerable group. Although not our focus, it is worth noting that stigma embedded in legal practices may also have clinical implications, influencing risk assessments and treatment pathways for defendants with mental health conditions.

In this intersection of criminal stigma and mental health stigma, the media play a significant role in shaping public perceptions and attitudes, often alongside political narratives. As we will return to in Sections 4 and 5, the intersection of criminal justice and mental health is increasingly framed in terms of risk and social safety (Gröning and Dimitrova, 2024). Previous research suggests that the media contribute to negative stereotypes portraying people with mental health conditions as ‘dangerous’ (e.g., Klin and Lemish, 2008; Smith, 2015; Manago et al., 2019). It has further been suggested that the idea of unpredictability and dangerousness has had the strongest impact on the desire for social distance desired toward individuals diagnosed with schizophrenia, and the more this view is adopted, the higher the desire for social distance (Angermeyer and Dietrich, 2006). This is noteworthy, because the diagnosis of schizophrenia is associated with legal notions of insanity and unaccountability in many criminal justice systems (Crocker et al., 2015; Tsimploulis et al., 2018; Gröning, 2025; see also Section 4). Although the dynamics of intersectional stigma are complex (Turan et al., 2019), the literature exploring this complexity remains limited (Johansen and Møllerhøy, 2016, p. 382). The intersection between mental health stigma and criminal justice—particularly regarding legal responses to defendants with mental health conditions—thus remains largely unexplored, possibly reflecting broader societal attitudes towards this group.

### 2.2 Comparing legal responses to defendants with mental health conditions

Our comparative investigation adopts a legal-doctrinal and analytical perspective, examining the special rules governing criminal responsibility and preventive measures for defendants with mental health conditions. It is centred on formal legal sources, examining both general rules and their application in practice. Hence, the central material consists of written legal texts, including codified rules, legislative proposals, court judgments. More specifically, we focus our investigation on the legal premises and arguments contained in these sources, which provide the criminal law’s authoritative public justifications for decisions on criminal responsibility, punishment, and compulsory treatment. Because the legal materials originate from different languages and legal traditions, the translation of legal terms has been undertaken with close attention to each system. Nonetheless, our English-language discussion cannot fully capture the nuances contained in the original terminology.

When discussing how legal responses to defendants with mental disorders relate to stigma, we draw on previous research and on our knowledge of the legal systems and legal-cultural contexts of the three countries. Our comparative investigation here combines functional, structural and law-in-context approaches (for these different approaches, and their possible combination, see, e.g., Van Hoecke, 2015 and Siems, 2022). We do not intend to make empirical claims but rather to provide a foundation for future empirical work.

As noted in the introduction, Bulgaria, Japan, and Norway share from our legal point of view, similar functions and structures in this area. Comparable arrangements are also considered to exist in many other jurisdictions (Mackay and Brookbanks, 2022). In this regard, we consider that there are some basic legal ideas about human agency and its limits that shape how legal systems approach defendants with mental health conditions (cf. Gröning et al., 2023). Across jurisdictions, these approaches address practical legal and societal challenges concerning the boundaries of criminal responsibility and the protection of social safety. At the same time, we acknowledge that the concrete rules and institutional organisation differ, reflecting legal culture, tradition, and broader societal context (Koch and Sunde, 2020; Lacey, 2016). As we will return to in Section 4, Bulgaria, Japan, and Norway illustrate substantial variation in this regard.

Previous research also suggests that stigma manifests differently across societies (Abdullah and Brown, 2011; Yang et al., 2013; Krendl and Pescosolido, 2020). In this regard, there are significant societal and legal-cultural differences between Bulgaria, Japan, and Norway that may influence the role and production of mental health stigma in the criminal justice context. While these differences cannot be described in detail in this paper, a brief outline of central features is needed as a backdrop for our further comparative investigation into legal responses to defendants with mental health conditions.

## 3 Societal and legal cultural differences between Bulgaria, Japan, and Norway

The societal and legal-cultural contexts of Bulgaria, Japan and Norway differ on many levels. Norway and Japan are constitutional monarchies, while Bulgaria is a Parliament Republic. Bulgaria and Norway have a relatively similar population size—Bulgaria with around 6,4 M inhabitants and Norway with around 5,5 M inhabitants. Japan has a population that is about 20 times larger with 124 million inhabitants, distributed over almost the same territory size as Norway. The inhabitants in these countries live under considerable different welfare and resource conditions. Bulgaria is one of the poorest countries in Europe, which is also reflected in its constantly decreasing population since 1989. Norway is on the other hand one of the richest countries in Europe and among the countries in the world with highest welfare (see further Gröning and Dimitrova, 2023). Japan is like Norway considered one of the richest countries of the world and has one of the world’s largest economies. At the same time, the Japanese welfare state has special features (Kitayama, 2024). It embodies familyism, where families rather than government provide the social safety net. It is usually assumed that in Japan resource allocation for personal social services for the elderly and disabled, and for government support of working mothers is far from European countries.

Moreover, religion plays different roles in Bulgarian, Japanese, and Norwegian societies. In Bulgaria, the dominant religion is Orthodox Christianity, but there are also religious minorities, such as a Muslim and Jewish minorities. Active religious engagement in Bulgaria is limited (National Statistical Institute Bulgaria, 2022). In Japan, most people are tolerant to religious beliefs, and many are simultaneously practicing several religions (syncretism). The Japanese government statistics: for 2024 show that the number of followers claimed by different religious are Shinto, 86.4 million, Buddhism, 80.5 milion, and Christianity 1.8 million (Statistics from Ministry of Education, Culture, Sports, Science and Technology, Japan, 2025). Noteworthy, the sum of these figures is nearly twice Japan’s population, a situation without parallel in other countries. (Official portal for the survey, statistical survey on religions of 2024). In Norway, religion plays a relatively modest role. Most Norwegian people are still affiliated with the Norwegian Lutheran Church, but the religious participation is low. There is an increasing religious diversity in the Norwegian society that include different Christian denominations, Catholicism and Islam (Statistics Norge).

Regarding legal culture and traditions Bulgaria and Norway are both European civil law countries. Norway belongs, however, to a specific Nordic legal culture (Greve, 2007; Sunde, 2021) that has often been associated with a particularly humane criminal law (Träskman, 2013; Pakes, 2020; Jacobsen, 2024). Current developments for dealing with defendants with mental health conditions challenge, however, this Nordic ideal (Gröning, 2024a). As we will come back to, these developments also have implications for mental health stigma (Section 5.3). In contrast, Japan is often considered to be a hybrid of civil law, common law and traditional Japanese customs (Kinoshita, 2001; Oda, 2021). While Japanese law is based on civil law influenced codified statutes, like Bulgarian and Norwegian law, the special rules for defendants with mental health conditions are common law based, although with unique features (Section 4.1). Moreover, considering the repressive character of the legal culture, the countries differ in terms of their maximum sentences. Japan retains the capital punishment for the most serious crimes, i.e., in practice in serious cases of murder, and practices executions by hanging. In January 2026, Osaka district court in this regard dismissed a request from three death-row inmates to ban such executions as they violate the International Covenant on Civil and Political Rights (The Japan Times, 2026). Bulgaria applies two forms of life imprisonment, with and without the possibility of parole, where the latter is used for the most serious crimes. Norway generally imposes a maximum prison sentence of up to 21 years, but allow for 30 years for war crimes, genocide, crimes against humanity and aggravated terrorism crimes. It must be noted, however, that Norway has a specific sentence of preventive detention (Norwegian penal code Section 40). This is time-unlimited imprisonment that can be prolonged based on considerations about social safety and danger of reoffending. These differences reflect how each country’s legal culture balances punishment, human rights, and public protection in distinct ways, showing a spectrum from Japan’s retention of capital punishment to Norway’s predominantly humanistic approach and time-limited sentencing philosophy.

The legal systems of the three countries have in this regard significantly different legal cultural and historical backgrounds (see further Gröning and Dimitrova, 2023; Oda, 2021). Norway has enjoyed a stable modern history as a constitutional democracy, shaped by social democratic principles that emphasise equality in healthcare, social services, and education (Reitan and Lien, 2023). In contrast, Bulgaria remained a satellite state of the Soviet Union until the collapse of its communist regime in 1990. Since then, the country has embarked on a path of progressive reform, steadily aligning its policies and institutions with EU legislation (Kostadinova, 2015). Still, there are some traces of the communist regime in current Bulgarian law. Of significance for our topic is that a crime in Bulgaria is defined as an “act dangerous to society” (Bulgarian penal code art 9). The Japanese legal system can be called a unique blend of multiple legal cultures (Oda, 2021). Japan’s modern legal system is largely rooted in civil law traditions from Germany and France. The current Japanese criminal law of 1907 is strongly affected by German law. After the second world war, Japan was also influenced by American, common law. This influence is visible in the current Code of Criminal Procedure that was promulgated in accordance with the principles of the new postwar Constitution in 1948 to fully protect fundamental human rights.

A significant difference between the countries as regards our topic concerns how the legal systems have been, and still is, shaped by different views upon the individual versus the collective. Many studies have emphasized that collectivistic and individualistic cultural orientations shape different experiences and expressions of mental health stigma (Yang et al., 2007; Ran et al., 2021; Ho et al., 2022). It has been suggested that people in collectivistic cultures tend to internalize mental health stigma more strongly than those in individualistic cultures (Yu et al., 2021). In these cultures, an individual’s mental health condition may, to a larger degree than in individualistic cultures, bring shame also to the individual’s family and community. Collectivistic cultures also tend to value social harmony, so behaviours that disrupt relationships or group peace are viewed more negatively than individualistic cultures (Krendl and Pescosolido, 2020; Kim and Markus, 1999). At the same time, cultural attitudes toward mental health vary within and across societies (see, e.g., Gopalkrishnan, 2018; Husain and Riasat, 2022). Especially Japan, but also Bulgaria is considered to have an emphasis on the collective. Both Bulgaria and Japan share a traditionally central role for the family. As we will return to below, this role of the family also raises special challenges in view of mental health stigma. Japan is in addition considered a paternalistic country, with strong emphasis on hierarchy and discipline in the society, which is deeply ingrained in every aspect of life. People are within Japan’s collectivistic culture implicitly expected to follow the suggestions, recommendations, or commands of the others, sometimes superiors, sometimes family members, rather than decide autonomously for themselves (Hayashi et al., 2000; see Sections 4, 5).

Norway, on the other hand, is mainly considered an individualistic culture that emphasises personal self-determination, individual responsibility and agency. Individual cultures are in this regard understood to have greater acceptance of individual variation, which then creates more favorable conditions for help-seeking behaviours and professional intervention acceptance (Kim and Omizo, 2003). The individualistic emphasis on self-determination and personal rights can serve protective functions against stigma. Norway’s individualistic culture may, however, create different challenges. People diagnosed with psychiatric disorders may for instance face stigma centered on personal failure of self-determination and individual inadequacy.

While individualistic cultures thus have been associated with more positive attitudes towards people with mental health conditions than collectivistic cultures, mental health stigma seems to relate to complex cultural factors. In our view, neither collectivism nor individualism should be treated as a uniform framework within the context of criminal justice and mental health. Still, the distinct cultural characteristics of Bulgaria, Japan, and Norway appear to create alternative legal pathways for addressing mental health stigma within the criminal justice context, which we will now turn our attention to.

## 4 The systems for handling defendants with mental health conditions in Bulgaria, Japan, and Norway

### 4.1 Special rules on criminal responsibility

Despite their societal and legal-cultural differences, Bulgaria, Japan, and Norway share several features in their systems for handling defendants with mental health conditions. All three countries stipulate, in their criminal codes, special rules on criminal responsibility for such defendants, which apply to individuals who are over the minimum age of criminal responsibility (MACR). The MACR is 14 in both Bulgaria and Japan, and 15 in Norway. None of the countries has specific rules on how mental health conditions should be considered for children (i.e., persons under 18) above the MACR (see, for challenges in Norway, Gröning et al., 2020; Øverland et al., 2026). In all three jurisdictions, the rules allow for acquittals where the defendant’s mental state at the time of the act exempts them from criminal responsibility. However, there are significant differences between the countries in how these rules are formulated and applied in practice.

Japan applies, like Anglo-American jurisdictions (see for an overview, Mackay and Brookbanks, 2022), an insanity *defence*. This means that the burden of proof rests on the defendants charged with a crime, to show that they were legally ‘insane’ at the time of the criminal act. This defence has a statutory basis in article 39 (1) of the Penal code of 1907 that stipulates that “Actions due to insanity is not subject to punishment” (Japanese law translation).^2^ This rule applies to individuals who at the time of the act could not distinguish right from wrong or could not control their behaviour due to their mental state (Japanese Supreme Court 1978.03.24 and 1983.09.13 Sono et al., 2008). The wrongness limb stems from the M’Naghten Rules used in English law (Lulu, 2025) requiring proof that the defendant did not know the nature of their act or not know that what they were doing was wrong.

It is extremely rare that defendants are acquitted by reason of insanity in Japan, as evidenced by the conviction rate that exceeds 99%. Most cases involving defendants with mental health conditions are not even prosecuted (see, e.g., Ministry of Justice, 2025), so that these defendants never get the opportunity to raise an insanity defence. There also seems to be a certain reluctance to raise this defence among prosecuted individuals. As we will discuss in section 5.2, this can be understood in light of the collectivistic culture and mental health stigma in Japan. At the same time, an insanity defense is an important way for defendants to avoid capital punishment in Japan, making its limited application a serious concern (Lulu, 2025).

Psychiatric evaluations in criminal proceedings are common in Japan, where the forensic experts commonly utilise the Diagnostic and Statistical Manual of Mental Disorders (DSM 5) published by the American Psychiatric Association. Since cases resulting in acquittal due to insanity are extremely rare, psychiatric evaluations are typically conducted during the prosecution phase. Taking the psychiatric evaluation into consideration, public prosecutors can decide not to file the case (Criminal procedure code, art. 247), which happens in most cases. This allows defendants with mental health conditions to be removed from the criminal justice system at an early stage and progress to medical treatment as defined by the Mental Health Welfare Act (see Sections 4.2 and 5.2). Moreover, there is large judicial discretion in insanity cases. Psychiatric evaluations suggesting that the defendant was ‘insane’ at the time of the act are commonly rejected by the court. There has also been a development in the last decades towards a narrower application of the insanity defense. In the 1980s, the diagnosis of schizophrenia influenced the application of this defense (Kashiwagi et al., 2020). Today, this is rarely the case, but defendants diagnosed with severe mental disorders are sentenced since the courts appear to place greater emphasis on the assessment of the defendant’s volitional prong—taking into account the defendant’s general circumstances and the nature of the offense—than on the presence or severity of a mental disorder (Lulu, 2025). Insanity acquittals, when they occur, are sometimes met with criticism from victims’ families or the public, who argue that the defendant should instead be subject to imprisonment or death penalty.

Norway and Bulgaria have very different systems than Japan. Here, it is the court that *ex officio* determines whether defendants should be acquitted because of their mental state at the time of the act. The burden of proof rests on the prosecution, and not on the defendant, to show that the defendant was criminally liable. Moreover, the prosecution has a duty to do an objective evaluation of all circumstances in the case. In these countries, more cases with defendants with mental health conditions are prosecuted and tried in the courts than in Japan, and many of these cases also lead to acquittals (Gröning and Dimitrova, 2023; Gröning and Dimitrova, 2024). However, as we will return to in Section 4.2, many of these defendants are subjected to special preventive measures.

In Bulgaria, the relevant rules governing criminal responsibility are set out in article 31 of the Bulgarian Penal Code, which provides the main rule that a person who has reached the age of 18 and who commits a crime while in a state of sanity is criminally liable. The Penal Code also establishes in article 33 (1) that responsibility does not apply to “a person, who has acted in a state of insanity (невменяемост)—where due to ‘retarded’ mentality or derangement of his consciousness of prolonged or short duration, the person has not been able to understand the nature and meaning of the act or to manage his actions” (official translation of the Bulgarian Penal Code).^3^

This rule is commonly considered to be based on a ‘mixed model’ incorporating both ‘medical’ and ‘psychological’ criteria (Gröning and Dimitrova, 2023). The medical criteria refer to certain impairments, exhaustively listed in art. 33 (1) of the Penal Code, that may lead to an acquittal. These include ‘mental retardation’ and ‘short or long derangement’. Experts are appointed by the court (or by the prosecutor in the pre-trial phase) to provide information about whether a defendant’s mental condition fulfils these criteria. Only the court, however, can make the conclusion of whether the defendant is ‘insane’ or not based on psychological criteria about the ability to understand nature and consequences of the act and to control one’s actions.

In Norway, the relevant rule about accountability (*skyldevne*) is provided by the Penal code Section 20. This statutory provision provides in its first paragraph the rule about the MACR (see further Gröning et al., pp. 494–498). The second to third paragraph of Section 20 provides rules about conditions other than low age that may imply that a defendant is unaccountable (see further Gröning et al., pp. 494–498).^4^ The determination of unaccountability requires a two-step evaluation. First, it must be considered whether the defendant’s condition fulfils one or several of the criteria specified in the first and second paragraph letters a-c. These are (a) a ‘severely deviant state of mind’—which includes psychotic states and equally serious mental states (cf., Supreme court judgments HR-2023-1242-A; HR-2023-1243-A), (b) ‘severely impaired consciousness’, cf. automatism, and (c), ‘severe mental disability’ with a guiding IQ-limit at 60 (cf. Supreme court judgment HR-2025-662-A).

Second, it must be evaluated whether defendants were ‘unaccountable due to’ their condition. In the third paragraph of the rule, it is prescribed that emphasis should in this evaluation be placed on the ‘degree of failure in the person’s perception in reality and functional capacity’. Forensic experts are appointed to inform the court about the mental state of the defendant, especially in view of these failures. It should be noted that, contrary to the law of most other jurisdictions—including Bulgaria and Japan—there is no legal requirement to assess whether the defendant’s condition influenced the commission of the crime (Legislative proposal, 154 L (2016–2017) pp. 13, 54–55).

Both in Norway and Bulgaria, forensic experts are appointed to inform the court through clinical and diagnostic evaluations. The experts in both countries commonly use the World Health Organization’s International Statistical Classification of Diseases and Related Health Problems (ICD-10) as a basis for their diagnosis of the defendant. In Norway, DSM 5 can also be used in the forensic context, although the primary recommendation is to use the ICD-system. The courts in both Bulgaria and Norway rely on the psychiatric evaluations of the experts (Gröning and Dimitrova, 2023). As we will return to in Sections 5.1 and 5.3, legal notions about insanity and unaccountability are associated with diagnostic categories, such as ‘schizophrenia’, which raises special concerns related to mental health stigma.

### 4.2 Preventive measures

In all three countries defendants acquitted because of their mental state at the time of act may be subjected to preventive measures, mainly compulsory treatment and confinement. The legal basis and the substance of these measures differ, however. In Bulgaria and Norway they are regulated as special criminal sanctions, which are formally not punishment, whereas in Japan, they are based on treatment orders issued by a panel at the district courts.

In Bulgaria, the grounds and procedures for sentencing a defendant to a special criminal sanction are listed in the Bulgarian Penal Code articles 89–92 and articles 427–432 of the Bulgarian Penal Procedure code. There are different types of compulsory measures that can be used, as exhaustively listed in article 89 of the Penal Code. These measures involve compulsory treatment in special psychiatric hospitals or wards or transferring the defendant to next-of-kin under mental health center supervision. The option of placement under next-of-kin means that the relatives or family need to take much responsibility for the supervision of the sentenced person and is commonly used in less serious crimes and to persons who are at law risk for themselves and the others. This next-to-kin placement is quite unique in international comparison and is culturally rooted in Bulgaria’s emphasis on the role of the family (Gröning and Dimitrova, 2024). While it may imply societal integration, public stigma in society often leads to isolation of the sentenced person (see Section 5.1).

To decide about the need for compulsory care, and what measures to use, the Bulgarian courts assess whether the defendant is ‘dangerous to society (Criminal Procedure Code, article 427, Section 2). Experts examine the mental condition of the defendant and inform the courts about what kind of treatment they need (inpatient or outpatient) and if the compulsory treatment will lead to an improvement of their condition. Bulgaria does not have any regulation about the maximum length of compulsory care, but the court determines its termination and continuation. The compulsory care must, however, be subjected to revision after 6 months (Penal code, art. 91). The court may cease or amend the type and length of the compulsory treatment when there is a change in the condition or treatment needs of the sentenced person. As we will return to in Section 5, there are many challenges in Bulgaria regarding the conditions for sentenced persons in compulsory treatments that are largely attributed to lack of resources.

In Norway, special criminal sanctions for unaccountable defendants are regulated in Section 62 and 63 of the Penal Code. According to these provisions, the court may decide to commit a defendant who is acquitted by reason of criminal unaccountability at the time of the act, to compulsory psychiatric care (Section 62) and compulsory care for mentally disabled persons (Section 63). Defendants who have committed serious crimes that violate life, health or freedom can be sentenced to time-unlimited compulsory care (Section 62, first paragraph). For defendants who have committed “repeated offences that are harmful to society or particularly bothersome”, but that do not violate life, health or freedom, the care can be limited to a maximum of 3 years (Section 62, second paragraph). This possibility of time limited compulsory care was introduced in 2016 (Legislative decision 46 (2015–2016)). Both forms of special criminal sanctions require a danger of re-offending and that the reaction is necessary to protect society. The time unlimited compulsory care can be prolonged as long as the danger for new serious crimes is considered to be present.

Compulsory psychiatric care is administered by the psychiatric hospitals according to the Mental Health Act (LOV-1999-07-02-62). There are no forensic hospitals for persons who are sentenced to compulsory care, but they are treated in general psychiatric hospitals. Compulsory care for mentally disabled persons is carried out in special units, but still within the general hospital structure. (see further in Gröning and Dimitrova, 2024). This organisation implies an important ethical view that no difference is made between patients who have committed a criminalised action, but were acquitted, and other group of patients. Every patient who needs mental health care should be treated equally, and be provided appropriate care, also considering preventing harm to others, on a civil law basis. In recent years, however, this view has increasingly been challenged. There is an increasing focus on social safety in the intersection between criminal justice and psychiatry. Defendants acquitted by reason of unaccountability are increasingly sentenced to time unlimited compulsory care because of the argued danger that they reoffend (Gröning and Dimitrova, 2024).

In contrast to Bulgaria and Norway, there are no regulations in the Japanese criminal code regarding compulsory admission and treatment of defendants acquitted by reason of insanity. The current regulation about such treatment is provided by the Act for Medical Treatment and Supervision of Persons with Mental Disorders Who Caused Serious Harm (subsequently, the Medical Treatment and Supervision Act; MTSA). This act came into force in 2005. Before this time, compulsory admission of defendants with mental health conditions was only possible under the Mental Health and Welfare Act (subsequently, MHWA). Under this legislation, defendants released from criminal justice proceedings were treated as involuntarily admitted patients in ordinary psychiatric hospitals alongside other psychiatric patients. Until 2005, these forms of civil admission also applied to defendants with mental health conditions who had committed serious offenses; that is, these individuals were treated within the framework of general psychiatry in Japan (Nakatani et al., 2009; Fujii et al., 2014)—similar to how this is still done in Norway. At the same time, more than 80% of Japan’s psychiatric facilities consist of private hospitals and are insufficiently equipped, security-wise, to treat defendants with mental health conditions (Fujii et al., 2014).

It was to change this situation, that the MTSA was passed in 2003 and came into effect in 2005. The aim of the act is to promote the rehabilitation of defendants who committed serious offenses while in a state of insanity or diminished capacity, reflecting current trends toward normalization and deinstitutionalisation. To this end, the Act established specific rules for managing defendants with mental health conditions who have committed serious crimes—such as homicide, arson, robbery, sexual intercourse without consent, indecency without consent, including attempts, and injury—and provides them with appropriate, continuous medical treatment and government-supervised care. The establishment of the MTSA marked the true beginning of the forensic mental health services in Japan. However, significant challenges remain in the treatment and reintegration of defendants with mental health conditions who have committed serious crimes (see Section 4).

The judicial Process of the MTSA is, in brief, as follows. First, public prosecutors may request the application of the MTSA if they decide not to charge a person who has committed a serious offense, as described above, on the grounds of insanity or diminished mental capacity, typically based on the results of a pre-trial psychiatric evaluation. Alternatively, a defendant who has been acquitted or given a reduced sentence without imprisonment in an ordinary criminal trial due to criminal insanity may be referred to the MTSA process. All defendants are entitled to access the courts to ensure that their rights are protected.

Following referral by the public prosecutor, the district court orders a MTSA psychiatric evaluation of the referred defendants as part of the court process, which is conducted in a specialized inpatient setting and must be conducted within 3 months. The primary purpose of the MTSA psychiatric evaluation is to verify the presence of a psychiatric disorder, treatability, and factors impending the defendant’s reintegration into society. Noteworthy, the criteria for admission into the MTSA system do not involve any reference to defendants’ recidivism risk. Such references to risk were included at early stages in the legislation process leading up to the enactment of the MTSA system but were deleted from the final bill to not evoke ideas of measures taken to ensure the safety of the public. Of significance for this decision was historical legal experiences and fear and opposition to proactive detention of individuals with mental health conditions for the sake of public safety. At the same time, the MTSA system applies specifically to defendants with such conditions who have committed serious offenses (Fujii et al., 2014). Since the introduction of this system established a separation of forensic and other patients, it also reflects broader concerns about social safety.

In the district court an interdisciplinary panel consisting of a judge and a specially qualified psychiatrist is set up when the MTSA psychiatric evaluation is ordered. At the same time, a rehabilitation coordinator who is on the staff of the probation office is assigned to each referred individual.

While the MTSA aims to facilitate adequate treatment, it does not, however, fully comply with this aim. Due to insufficient interaction between the MTSA system and the criminal justice system, many defendants with mental health conditions do not get access to needed treatment. As we will discuss below (Section 5.2), there are also regional variations in the implementation of the MTSA, as well as problems of unjustified long hospitalisations.

### 4.3 Intermediate observations

Our explorations so far reveals notable similarities and differences in the legal responses to defendants with mental health conditions across the three countries These relate to the substantive rules on criminal responsibility, their application in practice, the rules and systems governing preventive measures, and the treatment and reintegration of affected defendants. At each of these levels there seems to be structural problems that not only affect this group of defendants but may also contribute to broader mental health stigma. These problems seem to intersect in complex ways with legal labelling, system organisation and resources, and cultural norms and values.

In the following Sections, we will discuss further how the different legal rules, systems and practices may produce or reinforce mental health stigma in Bulgaria, Japan and Norway. We outline the central challenges in each jurisdiction as understood through our legal lenses and experiences. On this basis, we will compare the three countries to discuss the extent to which the challenges relating to mental health stigma in the criminal justice context are shared or divergent.

## 5 Challenges relating to mental health stigma

### 5.1 Challenges in Bulgaria

In Bulgaria, mental health stigma in society is well-documented. Previous studies suggest that mental health conditions in Bulgaria traditionally are associated with societal rejection, fear, and stigma (Stoyanov and Nakov, 2023; Dimitrova et al., 2023; Dimitrova, 2025). In a 2015 survey, 37% of respondents stated that a mentally ill person possesses negative personality traits, even describing them as “dangerous and aggressive”. Another study indicates that the Bulgarian people view persons diagnosed with mental disorders as significantly different from other people, which makes them unpredictable and dangerous, and justifies their separation from the society (Dimitrova et al., 2023). While public stigma is clearly present in Bulgaria (Marinova, 2020), there is at the same time a substantial lack of knowledge and awareness about mental health conditions among the general population, who are largely influenced by negative portrayals of people with such conditions in the media, news, and films (Dimitrova et al., 2023). There are no studies about mental health stigma related to legal responses to defendants with mental health conditions, although there are several challenges in this context that require attention.

An overall issue concerns the terminology used in rules governing criminal responsibility. The language in these provisions sometimes includes outdated terms (e.g., references to “retardation”) which also seem to legitimise stigmatising language in broader societal discourse about defendants with mental health conditions. Additionally, these rules often refer to diagnostic categories that no longer align with contemporary psychiatric understanding of mental health conditions as varying and responsive to treatment rather than as fixed states. In practice, courts, influenced by forensic expert testimony, sometimes link legal criteria for insanity to specific diagnostic categories like schizophrenia or bipolar disorder (see, e.g., SGC District Court Decision No. 225 of 18.11.2019). This may communicate stigmatising connections between these psychiatric diagnoses and criminal conduct (Kanushev, 2020). In addition, such practice fails to capture the diverse nature of mental health conditions and how they affect different individuals in different ways.

A striking practical challenge concerns the resources in the legal and forensic systems. Bulgaria faces severe resource limitations in its mental health infrastructure (Gröning and Dimitrova, 2024) that fundamentally shape how the legal and forensic system handles defendants with mental health conditions. Bulgaria’s mental health budget remains critically underfunded, with psychiatric services receiving disproportionately low allocation compared to other medical specialties. To some extent, this underfunding of mental health seems to relate to deinstitutionalization policies in Bulgaria in recent decades (Germanov, 2021). While these policies have reduced the funding of psychiatric institutions, they also seem to have led to a more general reduction in investment in mental health care in the community. Like many post-communist countries, Bulgaria has been under pressure to move away from large psychiatric institutions toward community-based care, in line with EU standards. However, this shift has occurred without the necessary investment in community mental health infrastructure.

The shortage of adequate funding creates a cascade effect: insufficient inpatient beds, outdated facilities, and limited community-based mental health services. This financial crisis forces the criminal justice system to become a “default” mental health provider (Nakov and Dimitrova, 2022). In our view, this is a clear example of structural stigma. Serious concerns are the critical shortage of forensic psychiatrists and trained mental health professionals specialising in criminal justice. This resource scarcity creates a stigma amplification cycle, i.e., lack of capacities seems to be a ‘driver of stigma’ at the same time as this lack of capacities may be explained by attitudes towards mental health.

Bulgaria also experiences prolonged institutionalisation for some patients who lack family support or adequate housing. Insufficient community resources have the consequence that patients remain in secure facilities longer than therapeutically necessary, reinforcing public perception of permanent dangerousness (Gröning and Dimitrova, 2024). Such perceptions are already strongly present in the Bulgarian society. Previous studies indicate that a clear majority of citizens think that psychiatric patients are dangerous to society and should not be allowed to roam freely in society (Kozhuharov et al., 2015). Media also frequently emphasize high rates of violence among individuals with mental health conditions, reinforcing negative stereotypes and misinformation (Martinova and Dimitrova, 2020). This may in turn influence criminal justice policies making the possibility of reintegration of sentenced individuals with mental health conditions even more challenging. In this context, the role of political discourse in shaping public stigma must also be noted. Sometimes, when the media report on serious criminal cases, Bulgarian politicians and policymakers comment publicly on them, often emphasizing the need for social safety and preventive measures.

In addition, resource limitations in Bulgaria influence the system to effectively rationing treatment based on perceived risk rather than therapeutic need. Criminal sanctions nominally aim at treatment but prioritise containment. Special criminal sanctions for defendants with mental health conditions in this regard rely primarily on the idea that violent behaviour can be treated, but resource constraints and security framing undermine this goal. This system design may harm vulnerable individuals and constitute a form of structural violence.

A special concern in Bulgaria is the lack of information about the situation for persons sentenced to special criminal sanctions. There is no public data about how many of these persons are treated in inpatient wards (this lack of data is also reflected in National Strategy for Mental Health 2021–2030). At the same time, it seems clear that the inpatient ward of the psychiatric hospital attached to the Lovech prison cannot accommodate all these people but many of them are treated in a general psychiatric hospital. The length of stay of these patients is usually long, and they also are considered to pose a greater danger to other patients and staff (National Strategy for Mental Health 2021–2030, p. 9). Such emphasis on danger also in this context often leads to prolonged institutionalisation due to a lack of community-based resources or lack of family support.

A special matter in Bulgaria concerns precisely the role of the family in the control and treatment of defendants sentenced to special criminal sanctions. Families play a crucial role in the control and treatment of these individuals. This can involve challenges considering that mental health is still a taboo in Bulgarian society, not only at the individual level but also at the level of the families and in the broader social context (Stoyanov and Nakov, 2023). Here, the public stigma surrounding mental health may also burden families, amounting to stigma by association. Families may face discrimination, judgment, or ostracism, both from their communities and their extended families which can negatively influence their engagement with mental health services or criminal justice systems due to fear of being labeled or judged. This in turn can result in inadequate support for sentenced individuals, potentially worsening their mental health and leading to recidivism.

The Bulgarian government has recognized the need to reform country’s mental health care system. To this end, Bulgaria has adopted the National Strategy for Mental Health 2021–2030, which focuses on promoting well-being, preventing illness, reducing stigma, and improving access to community-based care. However, the challenges relating to legal responses to defendants with mental health conditions are not specifically addressed in these plans.

### 5.2 Challenges in Japan

Mental health stigma is a significant challenge in Japan. Evidence of broader disability-related stigma appears in the (Cabinet Office of Japan, 2025) Public Opinion Survey, which includes people with mental and physical disabilities; 88.5% of respondents reported that discrimination and prejudice against persons with disabilities exist in Japanese society. Research also suggests that stigmatising attitudes toward people with mental disorders in Japan are stronger than in Taiwan or Australia, possibly due to long-standing institutionalisation, the absence of national anti-stigma campaigns, and a strong emphasis on social conformity (Ando et al., 2013). Japan’s focus on the collective makes stigma by association a specific challenge. A common perception is that having a family member diagnosed with schizophrenia can hinder marriage prospects.

Mental health stigma in Japan poses significant challenges for the criminal justice system and mental health services for defendants with mental health conditions (Fujii et al., 2014). As explained above, the insanity defense is rarely applied in Japanese courts, as prosecutors often choose not to pursue charges against defendants with mental health conditions. As a result, these defendants are deprived of fair and equal treatment under the law which in our view clearly amounts to structural stigma. In addition, public stigma—shaped in part by the way the media frequently report on crime—may discourage defendants from invoking this defense. Some may even prefer a criminal sentence over being legally classified as ‘insane.’ As in Bulgaria, the labeling of defendants as ‘insane’ carries potentially stigmatizing effects.

An interesting, related point is that the Japanese term for “schizophrenia,” tougou-shitchou-shou (“integration dysfunction disease”), was introduced in 2002 to replace the earlier term seishin-bunretsu-byo (“mind split disease”), which had increasingly been viewed as contributing to stigma. This change was initially requested by the National Federation of Families of People with Mental Illness (Zenkaren), which argued that the old term undermined a person’s dignity. After extensive deliberation, the Japanese Society of Psychiatry and Neurology approved the change in August 2002 and urged major media organisations to adopt the new term. Since then, tougou-shitchou-shou has become standard in medical contexts as well as in general public use. In our view, a similar revision of legal terminology is needed.

To the extent the insanity defense is applied in Japanese courts, it is rarely successful. Defendants with mental health conditions are commonly sentenced, even with capital punishment. This sometimes involves defendants who may not understand the situation they are in. While this situation compromises fundamental principles of criminal justice, it also has cultural explanations. It reflects a society where duty and collective shame are present and where mental illness is considered a matter of personal responsibility and weakness of character (Ando et al., 2013; Shiina et al., 2024). More specifically, discipline is considered one of the most important values in the Japanese society. People generally expect an apology and a remorseful attitude from those who fail to comply with social norms (Kashiwagi and Hirabayashi, 2018). These cultural norms impact the role and expectations of prosecutors to “succeed” in getting defendants punished.

Moreover, there is a strong sense of retributive justice regarding illegal acts. According to a Cabinet Office poll conducted in 2024, 83.1% of respondents answered that the death penalty is unavoidable, while only 16.5% expressed that it should be abolished (Cabinet Office, 2025). When a person who has committed a crime is not prosecuted or is acquitted due to insanity—and therefore does not express remorse or offer an apology—public opinion tends to align with the victim or the bereaved family, often calling for harsher punishment. A lack of remorse is rather often viewed as an indicator that rehabilitation may not be possible (Kashiwagi and Hirabayashi, 2018). Previous studies indicate that Japanese citizens are significantly less accepting of the insanity defense than those in the UK, and that this defense is considered a way to escape punishment (Shiina et al., 2024). A significant challenge in the Japanese system, as mentioned in Section 4, is the lack of a diversion mechanism between the MTSA and the criminal justice system. As a consequence, defendants with mental health conditions who are deemed fully responsible, or who have not committed a serious offence, are excluded from treatment. These persons serve their sentences in prison or medical prison without access to needed treatment. This is problematic, especially since most defendants with mental health conditions are sentenced, and there are no data on post-release outcomes. Furthermore, the diversion between the MTSA and the criminal justice system results in vastly different support systems for continuing medical care in community, despite its importance for preventing recidivism (Hirano, 2023).

Another aspect of these treatment challenges is their uneven regional implementation. Not all regions have MTSA inpatient facilities, resulting in regional variation in the availability and quality of services (Ministry of Health, Labour, and Welfare, 2025). When a patient’s post-discharge living arrangements is located far from the designated inpatient medical facilities, discharge planning during hospitalization becomes difficult. This sometimes leads to prolonged hospitalization due to societal risk aversion and a lack of community resources.

Long term hospitalisation within the MHWA system in Japan can also be related to the burden, and to stigma by association, on the family members of the psychiatric patients. These patients sometimes behave disruptively and have difficulty adjusting to their surroundings. It is often taken for granted that family members should care for and educate them to behave appropriately within the household, in line with family-centered care principles, which can place an excessive burden on the family. As a result, not only patients who lack support but also their family members may become isolated within the community. Moreover, when family members are unable to provide adequate care, this can lead to prolonged hospital stays, since problematic behaviour by a psychiatric patient is frequently regarded as the responsibility of the relatives who care for them (Sono et al., 2008).

In 2004, the Japanese government introduced a new mental health policy aimed at achieving deinstitutionalisation by further developing community-based services, encouraging hospital discharge, and reducing the numbers of beds. National health insurance, reviewed every 2 years, and medical plans, which determine the role and coordination of mental health services, continue to promote this direction. The 2024 amendment to the Mental Health and Welfare Act in 2024, which introduced a six-month upper limit on involuntary hospitalization and strict procedural requirements for extensions, is also important in this context.

Although the shift from institutional care to community living has thus progressed, institutional features often remain challenging in practice—for example, group homes located on hospital grounds or facilities that maintain strong supervisory control. Concerns have been raised about limited protection of service users’ self-determination, particularly for those coming from long-term hospitalisation. Japan’s strong cultural expectations of conformity may further complicate efforts of re-integration. Progress is also constrained by shortages in community-based resources. In many areas, key elements such as stable housing, outreach and home-visit services, peer support, and emergency-response systems remain insufficient. Deinstitutionalisation in Japan is thus moving forward slowly (Sugiura et al., 2024).

### 5.3 Challenges in Norway

There are several Norwegian studies that concern mental health stigma and how it affects people with mental health conditions (e.g., Simonsen et al., 2019; Rolandsgard et al., 2023; Waal and Clausen, 2022). While stigma is not considered a similarly serious societal problem as in Japan and Bulgaria, mental health stigma is a recognised challenge also in Norway. Stigma has been considered a specific challenge for people diagnosed with and treated for psychosis disorders (Simonsen et al., 2019). There is a lack of studies examining stigma related to criminal law mechanisms for handling defendants with mental health conditions, but concerns about stigma in this context have been raised (e.g., Bjørkly and Grøndahl, 2016; Gröning, 2017; SIFER report, 2023). These concerns are often about the association between mental health conditions and violent crimes that criminal law communicates and attitudes to this association that legal and psychiatric practices may involve.

Key challenges in Norway concern in our view how legal constructs of unaccountability and danger/dangerousness are associated with psychiatric constructs of mental disorders (Gröning, 2025). Legal criteria of “unaccountable due to” a “severely deviant state of mind” (Norwegian Penal code Section 20, third paragraph) are primarily associated with psychotic states (Supreme court judgments HR-2023-1242-A; HR-2023-1243-A). These criteria are also largely associated with diagnostic criteria, and commonly with the diagnosis of schizophrenia (Gröning et al., 2020; Gröning and Dimitrova, 2023). Moreover, the demarcation between diagnoses of a disorder in the psychosis spectrum, and diagnoses of personality disorders is considered legally important. According to the legal motives (Legislative proposal, Prop. 154 L (2016–2017) p. 76) and recent Supreme Court judgments (HR-2023-1243-A, paragraph 39), personality disorders should not be considered in unaccountability assessments. At the same time, such diagnoses are often linked to arguments about defendants’ risk of re-offending and are associated with notions about danger or dangerousness (HR 2026-167-A; Minderstrømmen, 2024). In recent years, the diagnosis of autism spectrum disorder has increasingly been emphasized as legally relevant (e.g., Norwegian Official Report, NOU 2014: 10, pp. 113–114; Legislative proposal, Prop. 154 L (2016–2017) pp. 76–77), potentially both for assessments of unaccountability and for evaluations of the risk of re-offending that may justify preventive measures. Both personality disorders and autism seem in this regard to be related to legal arguments that the defendant’s criminal behaviour depends on a disorder that will persist. A specific challenging area concern children who commit crimes and are diagnosed with autism. While this diagnosis rarely leads to unaccountability acquittal or reduction of the sentence, it seems to matter the more as a justification for indefinite preventive detention (Ducarre et al., 2025). This association between legal and psychiatric constructs is in our view also generally problematic, as the criminal justice system functions as a significant norm-setter in society (Gröning, 2024b). The interplay between the criminal justice system, the general population, political discourses, and media play an important role in this regard. Serious crimes committed by people diagnosed with mental disorders receive extensive media attention in Norway, which may contribute to fear and reinforce public mental health stigma.

Moreover, Norwegian criminal justice seems to be in a development towards increased emphasis on social safety and risk management, rather than proportionate and just punishment. In recent years, there has been a sharp increase in the number of unaccountable defendants who have been sentenced to special preventive sanctions. This indicates changing views about the role of criminal law responses to individuals with serious mental health conditions who cannot be held criminally responsible. This increased use of special criminal sanctions has been related to arguments that the civil psychiatry systems do not manage to prevent violent behaviour among people with mental health conditions to the extent society expects (Official Norwegian Report, NOU 2025: 2, pp. 210–211; Kilden et al., 2025). In addition, the increased use of criminal sanctions has been attributed to the increasingly restrictive legal grounds for the use of coercion in mental health legislation. While the threshold for using coercion in civil psychiatric care has been raised, a shift towards a “criminal justice psychiatry” with the “needed” coercive powers seems to occur (Gröning and Dimitrova, 2024). As a result of this development, psychiatric constructs of mental disorder seem increasingly to be connected to the criminal justice rationale of prevention and control, which may influence public attitudes to people diagnosed with such disorders as being dangerous, and produce or reinforce public stigma.

Noteworthy, it has been recognized in the Norwegian legal discourse that most people diagnosed with serious mental disorders do not pose any particular danger to others, and many who commit serious crimes do not have a diagnosis of a serious mental disorder. Mental disorder has been described as a possible risk factor for violence, especially in combination with other factors such as drugs, and access to good health care has been emphasized as significant to prevent people from developing violent behaviour (Official Norwegian Report, NOU 2025: 2, p. 278). At the same time, however, the tolerance in society towards the possibility of violent acts seems to have decreased over the last decade.

This risk focus in criminal law seemingly influences the treatment of unaccountable defendants who have been sentenced to special criminal sanctions of compulsory psychiatric. Principally, this patient group should not be discriminated based on their history of violence or other stigmatizing factors, and they should have access to municipal health and care services on equal terms with other citizens (Plan for Security Psychiatry, 2023; Bjørkly and Grøndahl, 2016). There seems, however, to be a development towards different legal approaches to the patients who have been sentenced to compulsory psychiatric care, and those who are committed on civil grounds. In this context, insufficient capacities seems like in Bulgaria to be a driver of structural stigma—despite the significant higher welfare level in Norway. In the Norwegian legal and mental health discussion, it is argued that the reduction in the number of inpatient beds has weakened the mental health services. When the hospitals are obliged to treat sentenced people, the result is that there are insufficient capacities to treat severely ill patients who have not committed any crime. These patients may in worst case develop violent and criminal behaviour, which in case may contribute to the increasing role of criminal law for people with mental health conditions (Official Norwegian Report, NOU 2025: 2, p. 280; Kilden et al., 2025). At the same time as legal and public attitudes about the need for social safety justifies special treatment of unaccountable defendants with such conditions, the knowledge basis for this special treatment is weak. There is limited knowledge about the effects of this treatment, and scarce systematized knowledge about how the special sanctions are carried out (this challenge is recognised in the Official Norwegian Report, NOU 2025: 2, pp. 33, 36–38).

There are also, like in Bulgaria and Japan, concerns relating to the reintegration in society for those sentenced to special criminal sanctions. These sanctions rely on the idea that violent behaviour can be treated so that the sanction eventually can be terminated and the individual be reintegrated into society. However, the current systems and practices do not fully manage to comply with this idea. A special practical challenge is the transition between inpatient treatment and treatment in the municipalities. Due to perceived lack of competence, resources, and adequate legal frameworks, especially in view of risk management, treatment in the municipalities can sometimes be difficult to realise. In addition, treatment can come with a significant financial burden. The result is then that some individuals are kept in psychiatric hospitals longer than necessary (Official Norwegian Report, NOU 2025: 2, p. 281).

The role of the family is much less formal in Norway than in Bulgaria and Japan, reflecting a more individualistic culture. In this regard, sentenced persons without family support may be more vulnerable to negative attitudes and mental health stigma in society, while stigma by association may be less pronounced than in Japan and Bulgaria.

### 5.4 Comparative discussion

Our comparative examination of Bulgaria, Japan, and Norway reveals distinct cultural-legal contexts and different legal responses to defendants with mental health conditions. It seems like stigma within the criminal justice context plays out in different ways in the three countries. Different views upon the individual versus the collective, and the role of the family, seems to be of significance. In Bulgaria and especially in Japan, stigma integrated in the collectivist culture seems to have influenced the legal systems to the disadvantages of defendants with mental health conditions. The extremely rare use of the insanity defence in Japan in our view demonstrates effects of discrimination and injustice for this group of defendants. Moreover, stigma by association also seems to burden family members to defendants with mental health conditions. In Norway, such collective mental health stigma does not seem to be equally present. The Norwegian context, however, shows how even well-resourced welfare states with reputations for humane criminal justice can drift toward risk-focused, control-oriented approaches when addressing mental health and crime.

Our findings suggest, however, that the challenges in the three countries cannot be fully attributed to cultural or societal characteristics. It appears that the legal systems in these countries do not merely reflect societal stigma; rather, they actively produce and reinforce it through mechanisms of labelling and separation, power asymmetries, and structural discrimination. Although the legal solutions in the three countries relate to stigma in diverse and complex ways, there are nonetheless striking commonalities among them.

The legal systems in all three countries apply rules on criminal responsibility using terminology that may have stigmatizing effects, not least in light of political discourses and media narratives surrounding criminal cases. Bulgaria’s Penal code employs both stigmatising notions of insanity and obsolete diagnostic language that legitimises a stigmatising public discourse. Norway’s legal criteria, while more modern, are still carrying negative connotations, such as of defendants with “severely deviant state of mind”, that are associated with broad diagnostic categories, thus also communicating a link between specific diagnosis and crime. Japan’s system, apply an ‘insanity’ defence, hence, even more explicit than Bulgaria, utilizing a label with clear negative connotations. The label ‘insanity’ has in this regard been criticised in the Anglo-American discourse for its negative and stigmatising connotations (Mackay and Brookbanks, 2022, p. 3).

Moreover, in all countries, legal notions of danger, dangerousness and violence shape the legal responses to defendants with mental health conditions, which may in turn reinforce public misconceptions about people with such conditions. Bulgaria’s incorporation of dangerousness of offenders with mental health conditions into its National Strategy for Mental Health represents a clear example, and similar approaches increasingly characterize Norwegian legal practices. In Japan, notions of danger and risk are not as explicit from legal rules, but social safety concerns still seem to be part of the rationale for special legal systems for defendants with mental health conditions. That criminal law employ labels that can contribute to public misconceptions about people with mental health conditions is a significant challenge, not least given the vital importance of language in shaping stigma (Thornicroft et al., 2022). Such labelling may also legitimise repressive criminal justice policies targeting defendants with mental health conditions. Societal portrayals of these defendants as particularly dangerous may justify intrusive preventive measures—and, conversely, the use of such measures may reinforce negative societal attitudes.

While it is recognised that deinstitutionalisation can facilitate stigma reduction (e.g., Winkler et al., 2021), achieving this appears to be challenging in this context. In all three countries, defendants with mental health conditions are subjected to special preventive control. Notably, in both Bulgaria and Norway there seems to be a shift towards institutionalization through the criminal justice system. An interesting point of comparison here concerns the separation between forensic and other psychiatric patients. Norway has still not implemented such a separation at the institutional level; instead, patients who have committed crimes are treated within the general hospital system. However, as discussed, this model is increasingly under pressure. Japan, by contrast, decided relatively recently—through the establishment of the MTSA system in 2005—to introduce such an institutional separation. Bulgaria also applies this type of separation, although with significant practical challenges due to insufficient capacity within forensic system.

In all three countries, however, some patients are kept longer in psychiatric and forensic institutions than can be justified as necessary and proportionate. This pattern is particularly troubling given that prolonged institutionalization may itself reduce prospects for community reintegration. This is in our view a serious problem of structural stigma that undermines human rights requirements related to the deprivation of liberty. This problem needs, however, to be seen in relation to how forensic and psychiatric services are organized. In all countries there are systematic challenges for needed and defensible treatment of defendants with mental health conditions. Japan’s lack of effective diversion between the MTSA and criminal systems creates gaps where these persons fall through, receiving neither adequate treatment nor appropriate criminal justice responses. Bulgaria and Norway face different but related challenges in the intersections between criminal justice and mental health systems, particularly during the transitions from institutional to community care.

Resource scarcity seems here to function as a driver of structural stigma in all three countries, although most evident in Bulgaria. To some extent, there seems to be a vicious cycle where insufficient resources are allocated to treatment of defendants with mental health conditions, with the result of insufficient rehabilitation and reintegration, further justifying their segregation and differential treatment. This may reinforce public fear and political pressure for more restrictive approaches.

Especially in Bulgaria, but also in Japan and Norway, insufficient community resources create barriers to reintegration—and the challenges of achieving deinstitutionaisation may also be linked to in adequate investment in community-based care. In Japan, cultural expectations of family care combined with stigma by association result in extended institutionalisation when families cannot or are not willing to bear such a burden. As a result, some persons remain in institutions instead of being reintegrated into society. A specific challenge in all three countries, particularly in Bulgaria, is the lack of systematic data especially which hinders evaluation and accountability.

In sum, our comparative legal investigation indicates that although patterns of stigma manifest differently across the three countries’ criminal justice contexts, they may operate through distinct legal pathways that ultimately produce similar stigmatizing effects. As such, our investigation indicates that stigma in the criminal justice context is not solely contingent on culture but is also produced and reinforced by the systems themselves through resource allocation, legal frameworks, institutional practices, and policy priorities that shape how defendants with mental health conditions are perceived. This raises the intriguing question of whether the underlying assumptions about human agency in modern criminal law also require closer scrutiny. Does mental health stigma stem, in part, from foundational legal ideas of the responsible agent? While a full examination of this lies beyond the scope of the present study, our findings suggest that the issue merits further attention.

## 6 The path forward

It is imperative to devote greater attention to the role of legal rules and practices in shaping mental health stigma in society. Our comparative investigation suggests that there are profound challenges across jurisdictions concerning how mental health and criminal stigma intersect, and how legal frameworks produce and reinforce stigma at this intersection. These dynamics include structural factors such as how legal frameworks labels defendants with mental health conditions, considerations related to resource allocation, and, importantly, the organisation of treatment systems.

In our view, there is an urgent need for legal reform that recognises the criminal justice system’s norm-setting role in society and its influence on public attitudes (Gröning, 2024a, 2024b, p. 23). Such reform should, we argue, include several elements. First, legal terminology in rules on criminal responsibility should be reviewed and updated to avoid reinforcing stigmatising associations between legal and psychiatric labels. Second, the challenges posed by structural stigma linked to the use of special preventive measures must be addressed. In this regard, systematic data collection, including the monitoring of outcomes for defendants subjected to such measures, is important. Finally, as we have argued elsewhere (Gröning and Dimitrova, 2024; Kikuchi et al., 2026), strengthening systems for community care and reintegration should constitute a central priority—one that requires dedicated and sustainable funding. In this context, the principle that criminal law should serve as society’s last resort—intervening only when less intrusive measures cannot prevent harm—must be reaffirmed.

The overarching challenge lies in identifying and transforming the institutional structures and practices within the criminal justice system that shape societal understandings of mental health. We have suggested that such an endeavor should address how criminal justice systems construct, categorise, and respond to defendants with mental health conditions. Such knowledge-based, legal reform is, however, a long-term project. It requires both and empirical inquiry, as well as data concerning the situation of people with mental health conditions within current criminal justice systems—both within and across jurisdictions. To this end, we hope that our comparative investigation and legal framework can stimulate further discussion and research within law and across other disciplines.

## Data Availability

The original contributions presented in the study are included in the article/supplementary material, further inquiries can be directed to the corresponding author.

## Footnotes

1 When referring to viewpoints, discussions, and legal discourses within the various national contexts, we will occasionally employ different terminology.

2 The original wording article 39(1) in its original Japanese language is as follows. is “心神喪失者の行為は、罰しない。». In addition, article 39 (2) provides a rule about diminished capacity that we will not discuss in this paper.

3 Article 33(1) of the Penal Code in its original Bulgarian language is as follows. “Не е наказателноотговорно лицето, което действува в състояние на невменяемост - когато поради умствена недоразвитост или продължително или краткотрайно разстройство на съзнанието не е могло да разбира свойството или значението на извършеното или да ръководи постъпките си.”

4 The rule in section 20, first to third paragraph, has the following wording in Norwegian: Den som på handlingstidspunktet er under 15 år, er ikke strafferettslig ansvarlig. Det samme gjelder den som på handlingstidspunktet er utilregnelig på grunn av. (a) sterkt avvikende sinnstilstand, (b) sterk bevissthetsforstyrrelse eller (c) høygradig psykisk utviklingshemming. Ved utilregnelighetsvurderingen etter annet ledd skal det legges vekt på graden av. svikt i virkelighetsforståelse og funksjonsevne.
